# Outcomes of Cesarean Myomectomies Performed at a Tertiary Care Hospital in North India: A Series of 10 Cases

**DOI:** 10.7759/cureus.79465

**Published:** 2025-02-22

**Authors:** Divya Mishra, Shravi Singh, Shantanu Shubham

**Affiliations:** 1 Obstetrics and Gynaecology, Graphic Era Institute of Medical Sciences, Dehradun, IND; 2 Neonatology, Graphic Era Institute of Medical Sciences, Dehradun, IND

**Keywords:** cesarean myomectomy, fibroid, myoma, myomectomy, pregnancy

## Abstract

Background

Uterine fibroids are common benign tumors in reproductive-aged women, and their prevalence in pregnancy is increasing. While cesarean myomectomy (CM) has traditionally been discouraged due to concerns about intraoperative hemorrhage and maternal morbidity, emerging evidence suggests it can be safely performed in selected cases. This retrospective case series evaluates the clinical outcomes of CM performed at a tertiary care center in North India over two years.

Methods

A total of 10 pregnant women who underwent CM were included in this study. Data were collected retrospectively from hospital records, including demographic details, indications for CM, fibroid characteristics, intraoperative parameters, and postoperative outcomes. Descriptive statistical analysis was used to summarize the findings.

Results

The mean maternal age was 34.5 years (range: 27-40 years), and the gestational age at delivery varied from 35 weeks and 4 days to 38 weeks and 6 days. The most common indication for cesarean section was fibroid-related concerns, including lower uterine segment (LUS) fibroids, multiple fibroids, and fibroids in patients with prior cesarean deliveries. Fibroid numbers ranged from one to fifteen per patient, with intramural fibroids (FIGO 4) being the most frequent type. The mean intraoperative blood loss was 620 mL (range: 450-800 mL), with no case of postpartum hemorrhage. The mean operative time was 74 minutes, and two patients required blood transfusions. No patients required intensive care, and the average hospital stay was 3.6 days. Neonatal outcomes were favorable, with a mean birth weight of 2.92 kg and reassuring APGAR scores. Three neonates required NICU admission for transient tachypnea, small for gestational age, and meconium aspiration syndrome.

Conclusion

CM, when performed in appropriately selected cases, is a feasible and safe procedure. It eliminates the need for a second surgery, reduces future fibroid-related complications, and does not significantly increase maternal morbidity. These findings support the growing consideration of CM as a viable surgical option in select cases. Further prospective studies are needed to establish standardized protocols for patient selection and surgical technique.

## Introduction

Uterine myomas, the most prevalent benign tumors in women, are increasingly being diagnosed in pregnant patients due to rising maternal age. Previous studies have reported a prevalence ranging from 0.1% to 3.9%, though the actual incidence could be higher, as some cases are only identified during cesarean section (CS) [1]. Every obstetric surgeon who encounters uterine fibroids during a CS is guided by the longstanding principle that fibroids are best left undisturbed during lower segment cesarean section (LSCS) [2]. Fibroids in pregnancy are not without challenges, as they can lead to various complications for expectant mothers. These may include postpartum hemorrhage (PPH), preterm labor, miscarriage, bleeding, and pressure-related discomfort due to hormone-induced fibroid growth. Additionally, degenerative changes can occur, with red degeneration being a notable concern. This particular type of degeneration, frequently observed in pregnancy, results from significant hemorrhagic infarction of the fibroid due to impaired venous drainage and thrombosis along its outer margins [3-5].

In 1914, Victor Bonney documented the first known case of cesarean myomectomy (CM). He successfully removed six fibroids during a CS performed on a 30-year-old woman having her first child. The procedure proceeded without complications, and the patient later had three subsequent vaginal deliveries [6]. A case series was published later on involving 13 patients who underwent CM, demonstrating its feasibility in carefully chosen cases [7]. However, concerns about the potential for severe, uncontrollable bleeding during the procedure led to its general reluctance among surgeons.

Performing a myomectomy during a CS offers several benefits. One significant advantage is that it eliminates the need for a separate surgical procedure, thereby reducing overall healthcare costs and minimizing the risk of fibroid-related complications in future pregnancies [8]. Additionally, removing fibroids at the time of CS promotes faster uterine involution during the postpartum period and lowers the likelihood of postpartum complications such as excessive menstrual bleeding, pain, and anemia [9, 10].

## Materials and methods

This retrospective case series was conducted at a tertiary care center in North India to analyze the clinical outcomes of patients who underwent cesarean myomectomy. The study included a total of 10 patients who were identified from medical records based on predefined inclusion and exclusion criteria. We included patients between January 2023 to December 2024, over a period of two years. The inclusion criteria comprised pregnant women who underwent CS with concurrent myomectomy and had complete clinical, intraoperative, and postoperative data available. For removal of myoma along with LSCS, one or both of the following criteria were used: Patients symptomatic for fibroid prior to pregnancy and/or presence of a fibroid measuring more than 15 cm in greatest dimension. All of these patients were either symptomatic for fibroids (posing a need for surgery) or had fibroids more than 15 cm in size and the likelihood of their significant shrinkage post-delivery was minimal. Patients with incomplete medical records were excluded.

Data collection involved extracting demographic, clinical, intraoperative, and postoperative details from hospital records. The recorded variables included patient demographics such as age, parity, and gestational age at delivery, as well as indications for CS and myomectomy. Fibroid characteristics, including number, size, and location were also documented. All of these patients were followed up for at least three months post-surgery. For short-term repercussions, we studied the incidence of postpartum hemorrhage, need for post-operative blood transfusion, post-operative drop in hemoglobin, ICU requirement and length of hospital stay. During subsequent follow-up patients were evaluated for uterine involution, general health, bleeding per vaginum and lochia, post-surgery infections and lactation.

For statistical analysis, descriptive statistics were employed to summarize findings. Statistical analysis was performed using STATA, Version 14 (StataCorp LLC, College Station, TX, USA). Continuous variables were reported as mean ± standard deviation (SD) or median with interquartile range (IQR) where appropriate, while categorical variables were expressed as frequencies and percentages. Ethical approval for the study was obtained from the Institutional Ethics Committee (IEC Approval Number: GEIMS/IRB/RP/16/2025). Patient confidentiality was strictly maintained following institutional and ethical guidelines. Informed consent was taken from the patients. In our study, the informed consent process included a detailed discussion of potential risks associated with future pregnancies following cesarean myomectomy. These risks were explained to the patients as part of preoperative counseling and documented in the consent form. Specifically, patients were informed about the potential for uterine rupture in subsequent pregnancies, especially during labor, the possibility of requiring elective cesarean delivery in future pregnancies due to uterine integrity concerns, the increased risk of placental abnormalities such as placenta previa, placenta accreta spectrum disorders, and abnormal placental adherence, the need for close fetal and maternal monitoring in subsequent pregnancies to assess uterine scar integrity. Patients were counseled postoperatively regarding the appropriate interpregnancy interval, typically advised to be at least 18-24 months to allow for optimal uterine healing.

## Results

This retrospective case series included 10 pregnant women who underwent CM performed by the same surgeon. The mean maternal age was 34.5 years, ranging from 27 to 40 years. Among them, four were primigravida, while the rest had prior pregnancies. The period of gestation at delivery ranged from 35 weeks and 4 days to 38 weeks and 6 days. The most common indication for cesarean section was fibroid-related concerns, including LUS fibroids, multiple fibroids, and fibroids in patients with previous LSCS. Some patients underwent CM based on maternal request. The most frequently associated comorbidities were hypothyroidism (6/10 cases), intrahepatic cholestasis of pregnancy (IHCP) (4/10), gestational diabetes (3/10), and gestational hypertension (2/10) (Table 1).

**Table 1:** Demographic and clinical characteristics POG: Period of gestation; G: Gravida; P: Parity; A: Abortion; L: Live issues; LUS: Lower uterine segment; LSCS: Lower segment cesarean section; PPROM: Preterm prelabour rupture of membranes; MSL: Meconium-stained liquor; IHCP: Intra-hepatic cholestasis of pregnancy

Case	Age (Years)	Obstetric score	POG (Weeks+Days)	Indication	Comorbidities
1	27	Primigravida	37+2	LUS fibroid	None
2	34	G3P1L1A1	37+1	Large fibroid with previous LSCS	Hypothyroidism
3	31	G2A1	38	Maternal Request	IHCP
4	36	G2P1L1	35+4	Previous LSCS with PPROM	Gestational Hypertension, IHCP
5	34	G4P1L1A2	36+6	Multiple Fibroids	Hypothyroidism, Anemia (Corrected)
6	39	Primigravida	37+2	Multiple Fibroids, Maternal Request	Gestational Diabetes, IHCP, Hypothyroidism
7	33	G2P1L1	37+6	Large fibroid with previous LSCS	Hypothyroidism, Gestational Diabetes
8	35	Primigravida	38+6	MSL	Hypothyroidism, IHCP
9	36	G3P2L1	37+1	Previous 2 LSCS with fibroid uterus	Gestational, Hypertension, Gestational Diabetes, IHCP
10	40	Primigravida	36	Multiple Fibroids	Hypothyroidism

**Figure 1:**
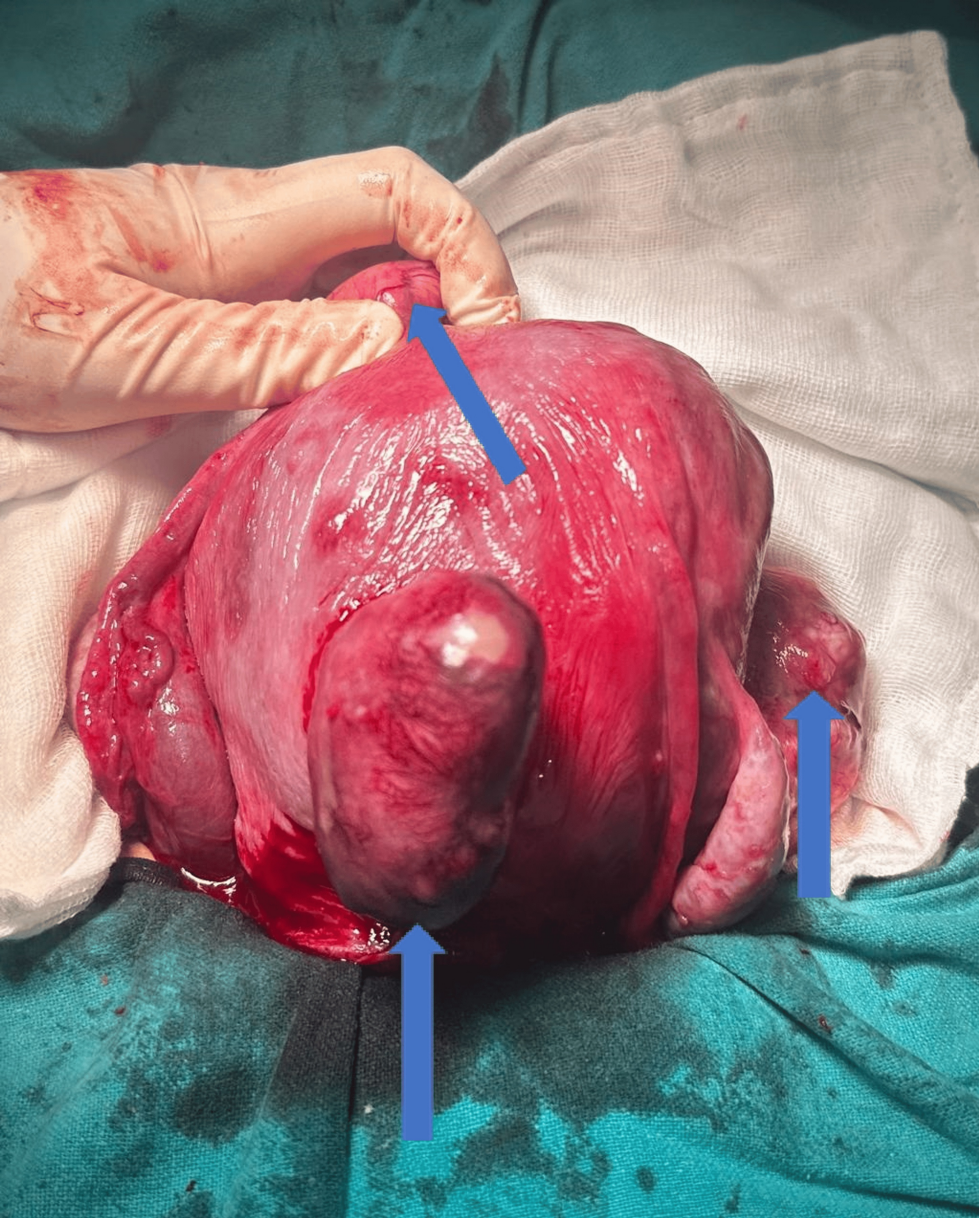
Multiple fibroids shown by blue arrows

The number of fibroids per patient varied from one to fifteen, with five patients having a single fibroid and five patients presenting with multiple fibroids (Figure 1).

**Figure 2:**
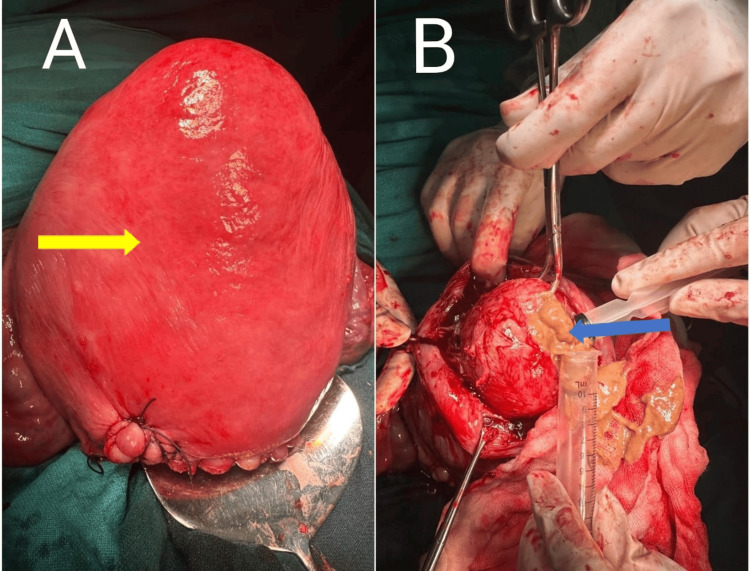
(A) Fundal fibroid (yellow arrow) and (B) Aspiration of necrotic material from the fibroid (blue arrow)

The most commonly encountered anatomical location was intramural fibroids (FIGO 4), seen in six cases. Other fibroid types included subserosal fibroids with varying intramural extension (FIGO 5-6), and one case had a pedunculated subserosal fibroid (FIGO 7). The largest fibroid measured 20×12 cm and was located in the left lateral uterine wall extending to the LUS. Degenerative changes were observed in three cases, two showing calcific degeneration and one showing necrotic degeneration (Figure 2, Table 2).

**Table 2:** Uterine myoma characteristics in pregnant women FIGO: International Federation of Gynaecology and Obstetrics; LUS: Lower uterine segment

Case Number	Number of Fibroids	Gross Location	Anatomical Location	Dimensions (cm)	Degeneration
1	1	Left lateral uterine wall extending up to LUS	Subserosal < 50% intramural (FIGO 6)	20x12	None
2	1	Fundo-posterior	Subserosal > 50% intramural (FIGO 5)	15x12	None
3	1	Fundal	Intramural (FIGO 4)	12x9	Calcific
4	3	Anterior wall	Intramural (FIGO 4)	9x5	None
		Right lateral extending to LUS	Subserosal < 50% intramural (FIGO 6)	8x6	None
		Left Cornual	Intramural (FIGO 4)	5x5	None
5	5	Anterior wall	Hybrid Myoma (FIGO 3-5)	8x8	Calcific
		Anterior wall	Subserosal < 50% intramural (FIGO 6)	5x4	None
		Fundal	Intramural (FIGO 4)	4x3	None
		Posterior wall	Subserosal < 50% intramural (FIGO 6)	3x3	None
		Posterior wall	Subserosal < 50% intramural (FIGO 6)	2x2	None
6	2	Fundal	Intramural (FIGO 4)	12x8	None
		Left Anterolateral	Subserosal > 50% intramural (FIGO 5)	8x6	None
7	1	Fundo-anterior	Subserosal > 50% intramural (FIGO 5)	11x7	None
8	3	Right Anterolateral extending up to LUS	Subserosal < 50% intramural (FIGO 6)	8x6	None
		Anterior wall	Pedunculated subserosal (FIGO 7)	3x2	None
		Fundal	Subserosal > 50% intramural (FIGO 5)	3x2	None
9	1	Fundal	Intramural (FIGO 4)	8x8	Necrotic
10	15	Anterior wall of LUS	Intramural (FIGO 4)	15x8	None
		Posterior wall	Subserosal < 50% intramural (FIGO 6)	8x5	None
		Fundo-posterior	Subserosal < 50% intramural (FIGO 6)	5x4	None
		Left Posterolateral	Subserosal > 50% intramural (FIGO 5)	5x3	None
		Right Cornual	Subserosal < 50% intramural (FIGO 6)	5x3	None
		Fundal	Subserosal > 50% intramural (FIGO 5)	4x3	None
		Fundo-posterior	Subserosal > 50% intramural (FIGO 5)	3x3	None
		Left Fundo-anterior	Intramural (FIGO 4)	3x3	None
		Anterior wall	Intramural (FIGO 4)	3x2	None
		Anterior wall	Subserosal < 50% intramural (FIGO 6)	3x1.5	None
		Fundal	Subserosal < 50% intramural (FIGO 6)	2.5x1.5	None
		Anterior wall	Subserosal < 50% intramural (FIGO 6)	2.5x1.5	None
		Anterior wall	Subserosal < 50% intramural (FIGO 6)	2x1.5	None
		Anterior wall	Subserosal < 50% intramural (FIGO 6)	2x1.5	None
		Anterior wall	Subserosal < 50% intramural (FIGO 6)	2x1	None

**Figure 3:**
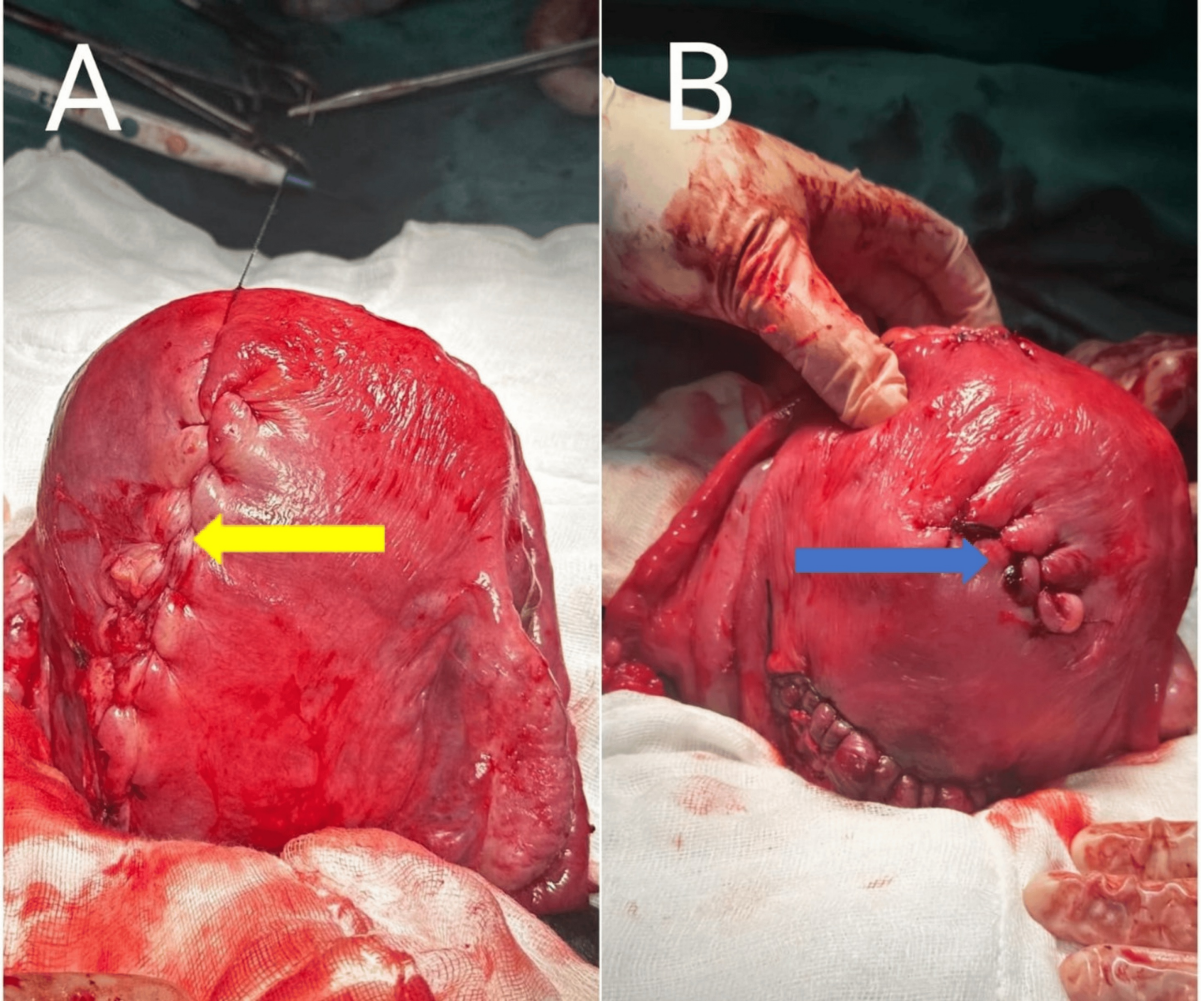
Final layer closure of myoma defect by baseball technique. (A) Posterior aspect (yellow arrow), (B) Anterior aspect (blue arrow) of the same uterus showing different myoma defects

Suturing of the myoma defect was done in layers using barbed sutures, with the final layer closure done by baseball technique (Figure 3).

The mean intraoperative blood loss was 620 mL, ranging from 450 to 800 mL. There were no incidences of PPH. The hemoglobin drop ranged from 0.8 to 2.3 gm/dL, with two patients requiring blood transfusions. The mean operating time was 74 minutes, with the longest case lasting 130 minutes, which involved enucleation of 15 fibroids. None of the patients required intensive care unit (ICU) admission postoperatively. The average length of hospital stay was 3.6 days, ranging from three to five days (Table 3).

**Table 3:** Intraoperative findings and investigational parameters Hb: Hemoglobin; PPH: Postpartum hemorrhage

Case	Pre-Op Hb (gm/dL)	Post-Op Hb (gm/dL)	Drop in Hb (gm/dL)	Blood Loss (ml)	Operating Time (minutes)	Transfusion	PPH	Length of Hospital Stay (days)	Need of intensive care
1	12.2	10.4	1.8	600	55	No	No	3	No
2	11.6	9.5	2.1	700	70	No	No	3	No
3	10.8	9.7	1.1	450	62	No	No	3	No
4	10.2	7.9	2.3	800	80	Yes	No	5	No
5	11.2	9.8	1.3	500	75	No	No	4	No
6	10.6	9.0	1.6	650	60	No	No	3	No
7	10.1	8.2	1.9	750	65	Yes	No	3	No
8	11.6	10.1	1.5	450	70	No	No	4	No
9	11.0	10.2	0.8	550	80	No	No	4	No
10	12.2	10.1	2.1	700	130	No	No	4	No

The neonatal outcomes were favorable overall. The mean birth weight was 2.92 kg, with a range of 2.20 to 3.35 kg. APGAR scores were reassuring in all cases, with all neonates scoring at least 7 at one minute and 9 at five minutes. Three neonates required neonatal intensive care unit (NICU) admission. The reasons for admission included preterm birth with transient tachypnea of the newborn (TTNB) and small for gestational age (SGA) in one case, and meconium aspiration syndrome (MAS) in another. One newborn had a calcaneovalgus foot deformity, but no major congenital anomalies were reported (Table 4).

**Table 4:** Details of baby APGAR: Appearance, pulse, grimace, activity and respiration; NICU: Neonatal intensive care unit; TTNB: Transient tachypnea of newborn; SGA: Small for gestational age; MAS: Meconium aspiration syndrome.

Case	APGAR 1 minute	APGAR 5 minutes	NICU Admission	Reason for NICU Admission	Birth Weight	Deformity
1	8	9	No	-	2.84	No
2	8	9	No	-	3.10	Calcaneovalgus Foot
3	9	9	No	-	3.26	-
4	7	9	Yes	Preterm, TTNB, SGA	2.20	-
5	8	9	Yes	Preterm, TTNB	3.10	-
6	7	9	No	-	2.90	No
7	8	9	No	-	3.15	No
8	8	9	Yes	MAS	3.35	No
9	8	9	No	-	2.70	No
10	8	9	No	-	2.68	No

## Discussion

CM remains a topic of debate due to concerns about intraoperative hemorrhage, postoperative complications, and overall maternal safety. However, recent studies, including this case series, demonstrate that in carefully selected patients, CM can be performed safely without significant morbidity.

In this study, 10 pregnant women underwent CM, with a mean maternal age of 34.5 years, aligning with prior reports suggesting that fibroids are increasingly common in older pregnant women due to delayed childbearing [11, 12]. The gestational age at delivery ranged from 35 weeks and 4 days to 38 weeks and 6 days, indicating that while fibroids can contribute to preterm birth, most patients delivered at near-term gestation [13]. The primary indication for cesarean section was fibroid-related concerns, particularly LUS involvement, multiple fibroids, and fibroids in patients with a history of previous LSCS. This finding is consistent with previous literature, which highlights that fibroids, especially in the LUS, may obstruct labor or increase the risk of uterine rupture, necessitating cesarean delivery [14].

The presence of comorbidities such as hypothyroidism (6/10), intrahepatic cholestasis of pregnancy (IHCP) (4/10), gestational diabetes (3/10), and gestational hypertension (2/10) is comparable to previous reports indicating that women with fibroids often have concurrent metabolic and endocrine disorders [15]. These conditions can further complicate pregnancy outcomes, making CM a viable option to prevent additional risks in future pregnancies.

In this study, fibroid characteristics varied, with numbers ranging from one to fifteen per patient. The most common anatomical location was intramural fibroids (FIGO 4), observed in five cases. Previous studies have shown that intramural fibroids can impact uterine contractility and increase the risk of PPH [16]. However, in this case series, no incidences of PPH were reported, and the mean intraoperative blood loss was 620 mL, which is comparable to or slightly higher than a standard cesarean section without myomectomy [17]. The use of injection vasopressin before myoma enucleation, along with the use of barbed sutures for myoma bed closure, likely contributed to effective hemostasis and minimal bleeding, as supported by earlier studies advocating advanced suturing techniques to reduce blood loss [18].

The mean operative time was 74 minutes, which is slightly longer than routine cesarean sections but within the acceptable range for combined procedures. The longest case (130 minutes) involved the removal of fifteen fibroids, reinforcing that operative time is influenced by fibroid number and location. Despite concerns about prolonged surgery leading to increased maternal morbidity, none of the patients required ICU admission, and the average hospital stay was 3.6 days, similar to other studies demonstrating that CM does not significantly prolong hospitalization when performed by experienced surgeons [19]. During subsequent follow-up, patients were evaluated for uterine involution, general health, bleeding per vaginum and lochia, post-surgery infections and lactation and these were comparable to patients undergoing only LSCS. Since these patients were either symptomatic for fibroids or had large fibroids, they would have required the removal of these fibroids prior to planning the next pregnancy. As the recovery was comparable to that in an LSCS, the fibroid removal should aid in preventing future miscarriages and infertility.

Neonatal outcomes were reassuring, with a mean birth weight of 2.92 kg and APGAR scores consistently above 7 at one minute and 9 at five minutes. Only three neonates required NICU admission, with causes including TTNB, SGA, and MAS. These outcomes are comparable to prior reports suggesting that fibroids may contribute to fetal growth restriction and respiratory complications due to uterine space constraints [20, 21]. Importantly, no major congenital anomalies were observed, aside from one case of calcaneovalgus foot deformity, which has not been directly linked to fibroids or CM in previous studies.

Overall, this study supports the growing body of evidence suggesting that CM, when performed in selected cases with appropriate surgical expertise, can be a safe and effective procedure. It eliminates the need for a second surgery, reduces future fibroid-related complications, and accelerates postpartum uterine involution, as also noted in previous research [22]. Concerns regarding massive hemorrhage, while valid, can be mitigated through meticulous surgical techniques, preoperative planning, and intraoperative hemostatic measures [23].

Future research should focus on larger prospective studies comparing CM with conventional cesarean sections to establish standardized protocols, particularly regarding patient selection, blood loss management, and long-term reproductive outcomes. Nevertheless, the findings of this study contribute to the evolving perspective that CM, rather than being universally avoided, should be considered a viable option in select clinical scenarios.

This case series has some limitations as well. The sample size is small, limiting the generalizability of the findings. As a retrospective study, it is subject to selection and reporting biases, with data being reliant on medical records rather than prospective analysis. Additionally, the absence of MRI imaging limits the precise characterization of fibroid size, location, and degeneration, which could have influenced surgical decision-making. The study does not include long-term follow-up data on reproductive outcomes or the recurrence of fibroids. Lastly, variations in surgical techniques and operator expertise may influence outcomes, requiring larger, multicenter studies for broader applicability.

## Conclusions

This case series demonstrates that CM can be a safe and effective procedure when performed in carefully selected cases. It eliminates the need for a subsequent surgery, reduces fibroid-related complications in future pregnancies, and does not significantly increase maternal morbidity. With meticulous surgical techniques and appropriate patient selection, concerns regarding excessive blood loss and postoperative complications can be mitigated. Favorable neonatal outcomes further support its feasibility. Larger prospective studies are needed to establish standardized guidelines and validate the long-term safety and benefits of CM in obstetric practice.
